# DPP-4 Inhibitor and Estrogen Share Similar Efficacy Against Cardiac Ischemic-Reperfusion Injury in Obese-Insulin Resistant and Estrogen-Deprived Female Rats

**DOI:** 10.1038/srep44306

**Published:** 2017-03-10

**Authors:** Sivaporn Sivasinprasasn, Pongpan Tanajak, Wanpitak Pongkan, Wasana Pratchayasakul, Siriporn C. Chattipakorn, Nipon Chattipakorn

**Affiliations:** 1 Cardiac Electrophysiology Research and Training Center, Faculty of Medicine, Chiang Mai University, Chiang Mai, 50200, Thailand; 2 School of Medicine, Mae Fah Luang University, Chiang Rai, 57100, Thailand; 3 Cardiac Electrophysiology Unit, Department of Physiology, Faculty of Medicine, Chiang Mai University, Chiang Mai, 50200, Thailand; 4 Center of Excellence in Cardiac Electrophysiology Research, Chiang Mai University, Chiang Mai, Thailand; 5 Department of Oral Biology and Diagnostic Science, Faculty of Dentistry, Chiang Mai University, Chiang Mai, 50200, Thailand

## Abstract

Estrogen deprivation aggravates cardiac injury after myocardial ischemia and reperfusion (I/R) injury. Although either estrogen or the dipeptidyl peptidase-4 (DPP-4) inhibitor, vildagliptin, reduces myocardial damage following cardiac I/R, their effects on the heart in obese-insulin resistant and estrogen deprived conditions remain unknown. Ovariectomized (O) rats (n = 36) were divided to receive either normal diet (NDO) or high-fat diet (HFO) for 12 weeks, followed by treatment with a vehicle, estrogen or vildagliptin for 4 weeks. The setting of *in vivo* cardiac I/R injury, 30-min ischemia and 120-min reperfusion, was performed. At 12 weeks after ovariectomy, both NDO and HFO rats exhibited an obese-insulin resistant condition. Both NDO and HFO rats treated with estrogen and vildagliptin showed reduced fasting plasma glucose, insulin and HOMA index. Both treatments improved cardiac function indicated by restoration of heart rate variability and increased %left ventricular ejection fraction (%LVEF). The treatments similarly protected cardiac mitochondrial function against I/R injury, leading to a reduction in the infarct size, oxidative stress and apoptosis in the ischemic myocardium. These findings demonstrate that vildagliptin effectively improves metabolic status, and shares similar efficacy to estrogen in reducing myocardial infarction and protecting cardiac mitochondrial function against I/R injury in estrogen-deprived obese-insulin resistant rats.

Cardiovascular disease (CVD) remains the major cause of mortality in both genders and accounts for 17.3 million deaths per year^1^. Coronary heart disease accounts for the highest percentage of deaths attributes to cardiovascular disease^1^. The incidence of CVD in women is lower than that in men, but this gender disparity is gradually reversed after the onset of menopause^2^. Estrogen deprivation from a bilateral ovariectomy (OVX) in women also causes an increase in mortality from CVD and an estradiol replacement can reduce the mortality risk indicating the impact of female sex hormone deprivation on heart problems^3^. Endogenous estrogen deficiency has been shown to result in more severe cardiac tissue damage and dysfunction after myocardial ischemia and reperfusion (I/R)^4^. In OVX animals which underwent cardiac I/R, cardiac mitochondria were damaged and disrupted to a greater extent than non-OVX animals, leading to decreased mitochondrial function when compared to intact ones^4^. Moreover, menopause is also associated with an increase in body weight, visceral fat weight, total cholesterol, LDL cholesterol, triglycerides and systolic blood pressure, all factors contributing to an increase in risk of metabolic syndrome^5^. The presence of metabolic syndrome could increase the risk of the development of cardiovascular disease, especially coronary artery disease and myocardial ischemia, which is the leading cause of mortality of women in many countries^6^.

Hormone therapy is an effective remedy for the management of menopausal-related problems such as bone health, reproductive tract symptoms, and vasomotor symptoms^7^. It also exhibited an advantage on prevention against metabolic syndrome and CVDs in estrogen-deprived individuals^8^,^9^. Although the benefits of estradiol replacement have been reported, its therapeutic effects are still being investigated. The use of hormone therapy is also limited due to the timing effect of hormone initiation^7^,^10^. Moreover, the hormone therapy is not recommended for menopausal women who have some health considerations such as reproductive cancers, thromboembolic disease, risk of stroke, as well as existent CVD or diabetes^7^. Therefore, alternative strategies for improving cardiometabolic function in estrogen-deprived individuals who have contraindications for estrogen therapy are still needed.

Vildagliptin is a member of the group of dipeptidyl peptidase-4 (DPP-4) inhibitor drugs. It is used for T2DM treatment. DPP-4 is a protease enzyme which inactivates glucagon-like polypeptide-1 (GLP-1) hormone, resulting in decreased insulin secretion. Thus, inhibition of DPP-4 helps in enhancing the GLP-1 level and increasing insulin secretion, thus lowering blood glucose level^11^. It has been shown that treatment with vildagliptin in high-fat-diet induced insulin resistant rats caused decreased cardiovascular oxidative stress in both heart tissues and plasma^12^. Vildagliptin also effectively restored heart rate variability to normal levels in obese-insulin resistant rats, indicating its positive impact in the regulation of cardiac sympathovagal balance. Moreover, cardiac mitochondrial morphology and function were completely restored in vildagliptin-treated insulin resistant rats^12^.

Both estrogen and vildagliptin also exhibited cardioprotective effects in instances of myocardial I/R injury by improving cardiac functional recovery, and reducing myocardial infarction and reactive oxygen species (ROS) production^4^,^13^. However, their protective roles on cardiometabolic function and I/R conditions had never been examined in obese-insulin resistant estrogen deprived rats. This study aimed to investigate the effects of chronic estrogen and vildagliptin treatments on the condition of obese-insulin resistance with estrogen-deprivation in combination with cardiac I/R injury. We hypothesized that estrogen and vildagliptin could improve metabolic status, cardiac function, cardiac autonomic regulation, and cardiac mitochondrial function and also inhibit oxidative stress and apoptosis in obese-insulin resistant estrogen deprived female rats who had undergone cardiac I/R injury.

## Results

### Efficacy of estrogen and vildagliptin on metabolic status

The estrogen-deprived rats demonstrated several features of obese-insulin resistance as indicated by significantly increased levels of body weight, fasting plasma glucose and insulin, plasma cholesterol and HOMA index in NDO group (Table 1). Plasma HDL level showed a significant reduction in NDO rats. The levels of body weight, visceral fat weight, fasting glucose, cholesterol and HOMA index showed a significant increase, and plasma LDL level was significantly increased in HFO rats, when compared with NDO rats suggesting the higher degree of metabolic disturbance induced by chronic high-fat consumption. Treatment with estrogen and vildagliptin similarly led to improved plasma glucose levels, insulin levels and HOMA index in both NDO and HFO rats. However, only estrogen treatment resulted in significantly reduced body weight in both NDO and HFO rats, and also significantly diminished visceral fat deposition in HFO rats. The levels of MDA were elevated in both serum and tissue samples from NDOVe rats, when compared with NDS rats which is the normal-diet fed sham rats, and these upregulated MDA levels in NDOVe rats were not significant different with HFOVe rats. Estrogen replacement and vildagliptin treatment led to significantly reduced serum and tissue MDA elevation in both NDO and HFO rats.

### Effect of estrogen and vildagliptin on cardiac function in the basal condition

**Figure 1:**
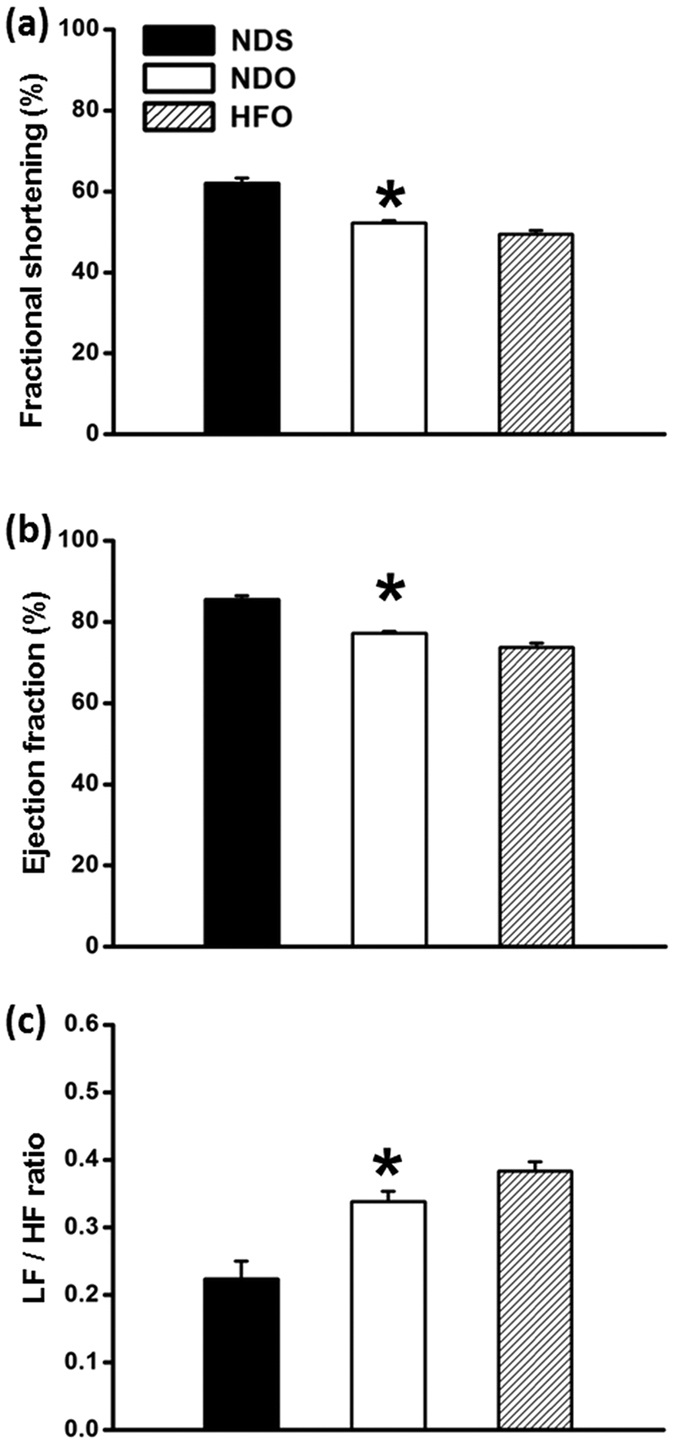
Effect of estrogen deprivation and obese-insulin resistance on left ventricular function and heart rate variability prior to pharmacological interventions. 12 weeks after ovariectomy, normal-diet fed ovariectomized rats (NDO) and high fat-diet fed ovariectomized rats (HFO) had reduced % fractional shortening (**a**), reduced % ejection fraction (**b**) and had increased LF/HF ratio (**c**), when compared with normal-diet fed sham operated (NDS) rats. Values are mean ± SEM for 6 rats in each group. *P < 0.05 vs. NDS.

**Figure 2:**
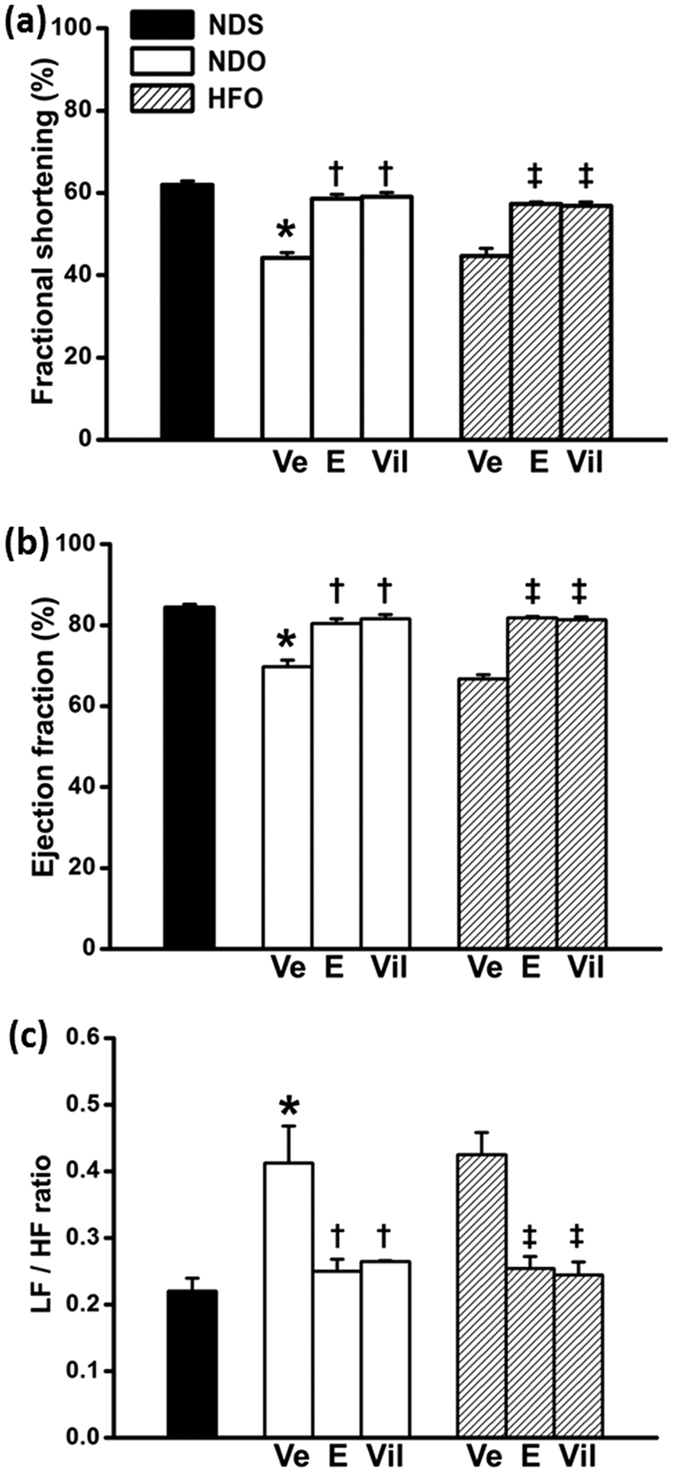
Effect of estrogen and vildagliptin on left ventricular function and heart rate variability of estrogen-deprived rats after treatments. % Fractional shortening (**a**) and % ejection fraction (**b**) were increased in estrogen (E)- and vildagliptin (Vil)-treated rats when compared with vehicle-treated rats. LF/HF ratio (**c**) was increased and restored to the normal level in both E- and Vil-treated groups. Values are mean ± SEM for 6 rats per group. *P <* *0.05 vs. NDS, ^†^*P *<* *0.05 vs. NDOVe and ^‡^*P *<* *0.05 vs. HFOVe.

Endogenous estrogen deprivation induced the impairment of cardiac function within 12 weeks, as indicated by decreased levels of %FS and %EF as well as depressed HRV in NDO rats (Fig. 1a–c, respectively). These parameters were not significant different between NDO and HFO rats. Both estrogen and vildagliptin treatments led to significantly increased %FS and %EF, and an improved HRV, when compared with vehicle-treated NDO- and HFO rats (Fig. 2a–c, respectively). Data from the P-V loop study indicated that LV function was impaired in vehicle-treated rats according to the lower levels of LVESP, ±dP/dt and SV/BW, and the higher level of LVEDP (Table 2). These cardiac function parameters were not different when compared between NDO and HFO rats. Estrogen and vildagliptin protected the heart from functional impairment by maintaining the levels of LVESP, LVEDP, ±dP/dt, and also increasing SV/BW ratio in both NDO and HFO rats, when compared with vehicle-treated rats (Table 2).

### Cardioprotective effect of estrogen and vildagliptin against cardiac I/R injury

#### Effects of interventions on LV function

Cardiac LAD occlusion induced a significant reduction in LVESP, −dP/dt and SV/BW in both NDOVe and HFOVe rats, when compared with NDS rats (Table 3). Furthermore, when compared with NDOVe rats, SV/BW ratio was further reduced in HFOVe rats. At the end of 30-minute ischemia, both estrogen- and vildagliptin-treated rats demonstrated a significant improvement in LVESP, LVEDP and SV/BW in both NDO and HFO rats, when compared with vehicle-treated groups (Table 3). Similarly to the ischemic period, NDOVe rats had significantly reduced LV function at the end of the reperfusion period as demonstrated by significantly decreased LVESP, increased LVEDP and lowered SV/BW ratio, when compared with NDS rats (Table 4). HFOVe rats demonstrated similar impaired cardiac function since there was no significant difference of the cardiac parameters, when compared with NDOVe rats. This impairment of parameters in both NDO and HFO rats was restored after treatment with estrogen and vildagliptin. Both estrogen and vildagliptin treatment led to significantly reduced LVEDP, increased LVESP and improved SV/BW in NDO- and HFO rats, when compared with vehicle-treated rats (Table 4).

#### Effects of interventions on cardiac arrhythmia and mortality rate

**Figure 3:**
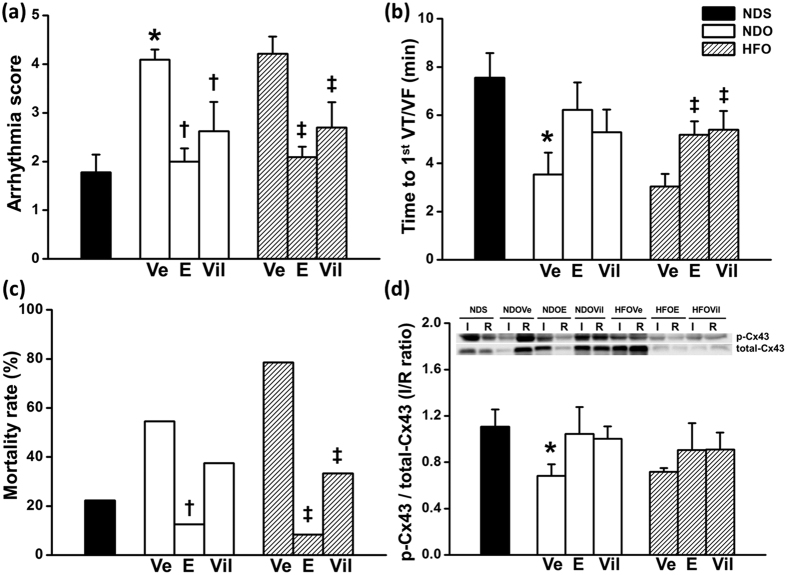
Cardiac arrhythmia, mortality rate and cardiac expression Cx43, after treatment with estrogen or vildagliptin for 4 weeks. Arrhythmia score (**a**) was decreased, and time to 1^st^ VT/VF (**b**) was prolonged in both estrogen (E)- and vildagliptin (Vil)-treated groups. Post-ischemia mortality rate (**c**), presented in % of number of rats in each experimental group was significantly reduced following treatment with E and Vil, when compared with vehicle-treated group. The ratio of p-Cx43/total-Cx43 expressions (**d**) in ischemic (I) area/remote (R) area, were reduced in NDOVE and HFOVe rats, when compared with those of NDS rats. Values are mean ± SEM for 6 rats per group. *P <* *0.05 vs. NDS, ^†^*P *<* *0.05 vs. NDOVe and ^‡^*P *<* *0.05 vs. HFOVe.

Arrhythmia score was significantly increased (Fig. 3a), and the time to 1^st^ VT/VF was significantly reduced in NDOVe and HFOVe groups (Fig. 3b). Treatment with estrogen and vildagliptin effectively reduced the arrhythmia score in both NDO and HFO rats (Fig. 3a). Both of the treatments also similarly delayed time to 1^st^ VT/VF in HFO rats, when compared with vehicle-treated rats (Fig. 3b). This increased mortality rate in HFO rats was significantly decreased following treatment with estrogen and vildagliptin (Fig. 3c). However, only estrogen treatment led to a significantly reduced mortality rate in the NDO rats, when compared with NDOVe group (Fig. 3c). The increased incidence of arrhythmia in NDOVe and HFOVe was consistent with the altered level of p-Cx43 in their cardiac tissue. The expression level of p-Cx43/total-Cx43 in ischemic areas/remote areas was significantly lower in NDOVe rats, compared to that in NDS rats (Fig. 3d), and the p-Cx43/total-Cx43 ratio was not significant different between NDOVe and HFOVe rats.

#### Effects of interventions on myocardial infarct size

**Figure 4:**
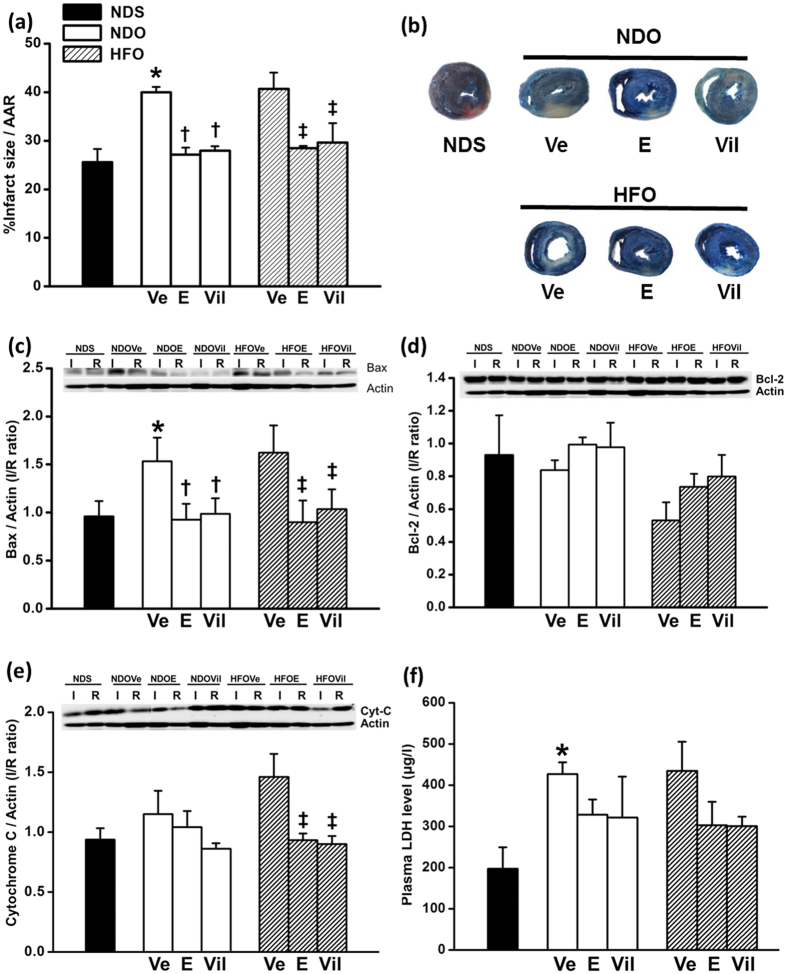
The levels of infarct size and cardiac expression of apoptotic and anti-apoptotic proteins, after treatment with estrogen or vildagliptin for 4 weeks. Both NDOVE and HFOVe had increased level of infarct size (**a**), and both estrogen (E)- and vildagliptin (Vil)-treatment significantly diminished myocardial infarction. The representative infarcted myocardium was demonstrated in (**b)**. Expression levels of the apoptotic protein Bax (**c**) were significantly increased in NDOVe and HFOVE rats, whereas the expression levels of anti-apoptotic protein, Bcl-2 (**d**) were reduced in HFOVe rats. The cardiac level of cytochrome C expression (**e**) was increased in HFOVe rats and significantly reduced by both E and Vil-treatments. Plasma LDH level (**f**) was significantly elevated in both NDOVe and HFOVe rats, when compared with NDS rats. The plasma level of LDH in the intervention groups was not significant different from the NDS rats. Values are mean ± SEM for 6 rats per group. *P <* *0.05 vs. NDS, ^†^*P* < 0.05 vs. NDOVe and ^‡^*P *<* *0.05 vs. HFOVe.

The levels of area at risk (AAR) were no different among the experimental groups. Data from the myocardial infarct size analysis showed that the percentage of infarct size per AAR was significantly increased in NDOVe and HFOVe rats, when compared with the NDS rats (Fig. 4a). Representative images of the infarcted myocardia are shown in Fig. 4b. The cardiac expression of apoptotic and anti-apoptotic proteins, Bax and Bcl-2, were investigated to confirm cardiomyocyte cell death, and we found that the expression level of Bax was significantly increased in NDOVe and HFOVe rats, when compared with NDS rats (Fig. 4c). Consistent with infarct size reduction (Fig. 4a), estrogen and vildagliptin treatment showed a significant reduced expression of Bax in the myocardium in both NDO and HFO rats (Fig. 4c). Moreover, HFOVe rats additionally demonstrated a significant decrease in Bcl-2 expression, when compared with NDS rats (Fig. 4d). The level of cytochrome C expression was found to be significantly increased in HFOVe hearts (Fig. 4e). Consistent with infarct size reduction, estrogen and vildagliptin treatment showed a significant reduced expression of Bax in the myocardium in both NDO and HFO rats (Fig. 4c), and also reduced cytochrome C expression in HFO rats (Fig. 4e). Plasma level of LDH was significantly increased in NDOVe and HFOVe (Fig. 4f), when compared with NDS rats, consistent with the upregulated Bax expression in these experimental groups.

#### Effects of interventions on cardiac mitochondrial function

**Figure 5:**
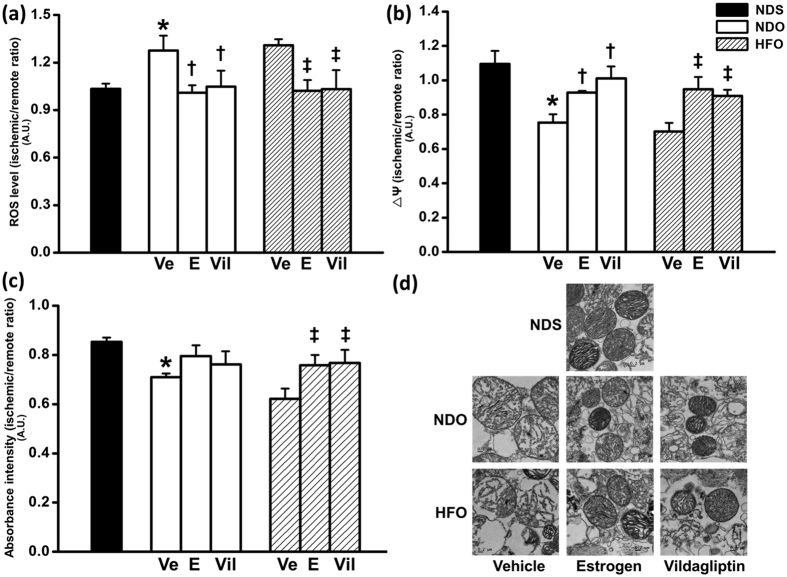
Cardiac mitochondrial function in estrogen-deprived rats after treatment with estrogen or vildagliptin for 4 weeks. All mitochondrial function parameters are represented in the ratio of ischemic/remote area. Mitochondrial ROS level (**a**) was increased while ΔΨ (mitochondrial membrane potential change, (**b**) and absorbance intensity (**c**) were decreased in NDOVe and HFOVe rats. Transmission electron micrographs (**d**) illustrate cardiac mitochondria morphology of rats in each group. Treatment with estrogen and vildagliptin led to an improvement in all parameters of cardiac mitochondrial function in both NDO and HFO rats. Values are mean ± SEM for 6 rats per group. *P <* *0.05 vs. NDS, ^†^*P* < 0.05 vs. NDOVe and ^‡^*P* < 0.05 vs. HFOVe.

After cardiac I/R, mitochondrial impairment was found in NDOVe and HFOVe groups. The parameters of mitochondrial function were assessed and expressed as an ischemic/remote area ratio. NDOVe rats demonstrated a significant increase in ROS production (Fig. 5a) and a decrease in mitochondrial membrane potential gradient (ΔΨ) (Fig. 5b), of the cardiac mitochondria in the ischemic/remote area indicating increased oxidative stress and mitochondrial depolarization, respectively. Absorbance intensity of the cardiac mitochondria from NDOVe rats was also lower than those of NDS rats (Fig. 5c), indicating mitochondrial swelling. These impaired mitochondrial parameters were not significance between NDOVe and HFOVe rats. Treatment with estrogen and vildagliptin resulted in a significant reduction in mitochondrial ROS production (Fig. 5a) and also a significantly increased ΔΨ in both NDO and HFO rats (Fig. 5b), when compared with NDOVe and HFOVe groups. The mitochondrial absorbance was restored to the normal level following both estrogen and vildagliptin treatments in HFO rats (Fig. 5c). The representative electron micrographs of cardiac mitochondria from the ischemic area of each experimental group are shown in Fig. 5d.

## Discussion

This study demonstrates the effect of estrogen deprivation with normal diet- and high-fat diet consumptions, as well as the efficacy of estrogen and vildagliptin, on metabolic status and cardiac function, during basal conditions and under conditions involving cardiac I/R injury. The major findings could be summarized as: (1) both NDO and HFO caused the impairment of cardiometabolic function in the basal condition; (2) when compared with NDS rats, the cardiac contractility and mitochondrial function were more diminished, and myocardial infarction and cardiac arrhythmias were more aggravated in both NDO and HFO rats underwent cardiac I/R injury; (3) chronic high-fat diet consumption aggravated the severity of cardiac I/R injury and increased mortality from cardiac I/R in HFO rats, and (4) an estradiol replacement and vildagliptin exhibited similar efficacy for cardioprotection against I/R injury in NDO and HFO rats.

Both estrogen deprivation and chronic high-fat diet consumption have long been known to be associated with the increased risk of metabolic dysfunction^14^,^15^,^16^. An increased level of oxidative stress has been previously revealed to be an important factor underlying the development of estrogen deprived- and obese-induced metabolic impairment^15^,^17^,^18^. In this study, the level of MDA was found to be elevated in both serum and cardiac tissues from both NDO and HFO rats indicating an increased level of oxidative stress in both peripheral circulation and the heart^19^. This finding emphasized the association between the elevated level of oxidative stress and metabolic disorders. Estrogen replacement and vildagliptin showed beneficial effects on the reduction of oxidative stress therefore could contribute to improved metabolic function in these rats. Consistent with a study in mice, estrogen provided anti-obese effects as it reduced body weight and visceral fat deposition in female rats^20^. This estrogenic action could be operated via lipogenic and lipolysis gene modulation, as well as may be involving central regulation via brain leptin and insulin sensitivity^21^,^22^.

Cardiac dysfunction indicated by cardiac autonomic imbalance, decreased levels of %EF and %FS, and impaired LV systolic and diastolic function, manifested itself after 12 weeks in both groups of rats. We have previously reported an association between endogenous estrogen-deprivation and an obese-insulin resistant condition and the development of cardiac dysfunction, involving the progressive increase of oxidative stress and cardiac mitochondrial impairment^15^. In this study, P-V loop analysis confirmed that LV dysfunction was already intensified in both NDO and HFO rats, when compared with NDS rats, in both a basal and I/R period. Moreover, myocardial dysfunction was more aggravated in HFO rats than in the rats that are estrogen-deprived alone. This is concluded from the marked reduction in stroke volume and increased LVEDP and mortality rate. A clinical study previously reported that coronary heart disease patients who had the higher level of homeostasis model assessment of insulin resistance (HOMA) index associated with the increased level of myocardial injury, as well as the higher risk of cardiovascular events and death, after receiving percutaneous coronary interventions^23^. Interestingly, the increased level of glucose intolerance and the higher HOMA index is associated with increased LV mass only in women^24^. This gender disparity may suggest that cardiac dysfunction in women is strongly related to and more amplified by metabolic disturbance.

Estrogen and its receptor activation have been reported as playing a pivotal role in cardioprotection via the regulation of oxidative stress against I/R injury^25^,^26^,^27^. Activation of estrogen receptor by its agonist during 1 week prior to cardiac I/R was demonstrated to reduce ROS production and attenuate inflammation through the reduction of TNF-α, IL-1β and LDH release^28^. Moreover, it has been shown that the expression of estrogen receptor in cardiomyocytes was increased under hypoxia condition suggesting the ischemic-induced upregulation of estrogenic-protective signaling pathway^29^. This cardioprotective effect of estrogen was consistent with the findings in the present study. In this study, depletion of endogenous estrogen initiated by ovariectomy as well as the combination of estrogen deprivation and high-fat diet consumption resulted in a higher level of cardiac oxidative stress as indicated by increased mitochondrial ROS production and increased myocardial MDA level. Increased mitochondrial ROS production is associated with mitochondrial membrane depolarization and mitochondrial swelling and subsequently triggered apoptosis^30^,^31^. This was indicated by increased Bax and cytochrome C expression^32^,^33^. It has been previously reported that consumption of a high-fat diet accelerates cardiac oxidative stress and cardiocyte apoptosis in an estrogen-deprived model without conditions of cardiac I/R^15^. In this study, NDO rats exhibited an increased level of cardiac Bax expression, whereas the HFO rats demonstrated an additional increase in cytochrome C, and decrease in cardiac Bcl-2 expression indicating the reduction of anti-apoptotic protein, therefore predisposing the cardiomyocytes to apoptosis during myocardial I/R injury. The increased level of cardiomyocyte apoptosis in both NDO and HFO rats was consistent with increased infarct size, when compared with NDS rats which had undergone I/R injury.

An estradiol replacement and vildagliptin showed their beneficial effects on the prevention of cardiac dysfunction in both basal conditions and during I/R injury in both NDO and HFO rats, as indicated by the preserved LVESP, LVEDP, ±dP/dt and SV. These treatments also decreased cardiomyocyte apoptosis and thus limited the size of myocardial infarction. According to previous studies, both estrogen and vildagliptin have been reported as protecting the cellular redox balance^34^,^35^,^36^. Treatment with estrogen has been shown to upregulate the level and activity of anti-oxidant enzymes (i.e. GSH/GSSG ratio, CAT, and SOD) in the myocardium, and also to decrease IL-6 and TNF-α level, suggesting it has anti-inflammatory effects in addition to anti-oxidative stress abilities^36^. Similarly, inhibition of DPP-4 activity by vildagliptin has been reported as reducing cardiac mitochondrial ROS production, therefore protecting mitochondrial function during conditions of I/R^37^. This effect of vildagliptin contributed to a decreased level of infarct size and a preservation of cardiac performance, which was diminished by I/R injury^34^. Moreover, treatment with vildagliptin also prevented the dispersion of cardiac electrical activity by inhibiting the shortening of the effective refractory period (ERP) in an I/R heart, resulting in a reduced arrhythmia risk^37^. In this study, the lower incidence of arrhythmia in both estrogen-treated and vildagliptin-treated rats was consistent with the maintained level of p-Cx43 expression in cardiac tissues, whereas the vehicle-treated rats demonstrated higher arrhythmia scores as well as decreased levels of cardiac p-Cx43 expression. This finding indicated that estrogen and vildagliptin provided cardioprotective effects against post-ischemic arrhythmia by maintaining Cx43 expression which contributes to the preservation of gap junction function and anti-arrhythmia effects^35^.

Estrogen and vildagliptin demonstrated similar protective efficacy on cardiometabolic status, mitochondrial function and also protected cardiac performance and prevented arrhythmia during I/R. These results suggested that vildagliptin may be prescribed to menopausal women who have contraindications for estrogen replacement therapy, in order to obtain similar benefits regarding cardiometabolic function. Vildagliptin had been demonstrated the similar therapeutic effects on cardiometabolic function in obese orchiectomized male rats with cardiac I/R injury condition suggesting that its efficiency was not influenced by gender discrepancy^38^. Its safety had been reported in several clinical studies to provide an effective glycemic control and could be used in patients who had renal and cardiovascular diseases, without producing renal risk and cardiovascular event^39^,^40^,^41^,^42^. Moreover, vildagliptin did not influence cardiac electrical activity since PR and QRS intervals were not altered during the prescribed treatment for T2DM patients^43^, therefore it could be a feasible alternative to estrogen therapy which had been known to associate with prolonged QT interval and QT dispersion in postmenopausal individuals who received long-term estrogen therapy^44^,^45^. However, our findings showed that estrogen treatment showed a significantly decreased mortality rate in both NDO and HFO rats, suggesting the superior efficacy of estrogen on the reduction of cardiac I/R-related mortality. This result was consistent with clinical reports which have demonstrated that hormone replacement therapy could reduce the incidence of coronary heart disease and total mortality, especially when initiated in recently postmenopausal women (less than 10 years following menopause)^3^. In this study, estradiol replacement was initiated at 12 weeks after endogenous estrogen deprivation, and demonstrated that it could effectively reduce the mortality rate in rats. From clinical studies in humans, the higher mortality rate in coronary artery disease patients and also over-all-cause mortality had been reported to be associated with a higher level of central obesity^46^,^47^. Therefore, the survival-promoting result of estradiol replacement may be owing to the body weight- and visceral fat-lowering effect, which are not found in the vildagliptin treatment group, in addition to its anti-arrhythmia, anti-oxidative stress and mitochondria protective effects.

In summary, findings from the present study indicate that both endogenous estrogen deprivation alone and combined estrogen deprivation with high-fat diet consumption caused cardiometabolic disorders and induced a higher degree of cardiac dysfunction (i.e. mitochondrial impairment, increased infarct size, contractile dysfunction, cardiac arrhythmia and higher mortality rate) following I/R, when compared with NDS rats. Although high-fat diet consumption aggravated the cardiometabolic status in an estrogen-deprived condition, it did not aggravate these impairments in the estrogen-deprived condition during cardiac I/R injury. The underlying mechanism of these cardiometabolic alterations could be associated with the increased level of oxidative stress caused by estrogen deprivation and metabolic disorders. Estrogen and vildagliptin exerted a similar efficacy on cardiometabolic improvement and also showed protection of the heart against I/R injury and attenuated post-ischemic arrhythmia resulting in a reduced mortality rate in these rats. The reduction of oxidative stress level in the heart and systemic circulation possibly plays an important role contributes to the potential effect of vildagliptin and estradiol treatments. However, further investigation is needed to completely clarify the proposed mechanism of vildagliptin and estradiol. Our findings provide new information for the application of this anti-diabetic drug, which confers cardioprotective benefits to menopausal women who have metabolic disorder and have contraindications for traditional estrogen replacement therapy. Future studies investigating the therapeutic efficacy of estrogen and vildagliptin for different periods and in various dosages are needed in order to warrant its use in a clinical setting in obese-insulin resistant menopausal women under condition of cardiac I/R.

## Materials and Methods

### Animals and ethical approval

Female Wistar rats (6 weeks of age, weighing 200–220 g) were obtained from the National animal center (Salaya campus, Mahidol University, Bangkok, Thailand). The rats were given time to acclimatize, and were housed in a temperature-controlled room (25 °C) with a 12-hour dark/light cycle setting. All experimental procedures were approved by the Institutional Animal Care and Use Committee at the Faculty of Medicine, Chiang Mai University, in compliance with NIH guidelines.

### Experimental protocol

Rats were randomly assigned to sham (S) or ovariectomized (OVX) group. The operations were carried out and then the rats were allowed to recover for 1 week. The sham group was fed on a standard normal diet (ND, containing 19.77% energy from fat), whereas the ovariectomized group was randomized and received either a ND or high-fat diet (HFD, containing 59.28% energy from fat) for 12 weeks^15^. At the end of the 12^th^ week, HRV and echocardiography were performed to record the pre-treatment data. OVX rats in both diet groups (NDO and HFO) were randomly subcategorized to be treated with an estradiol replacement (Estradiol, E; 50 μg/kg BW; daily, via subcutaneous injection), vildagliptin (Vil; 3 mg/kg BW; daily, via intragastric gavage) or sesame oil (as a vehicle, Ve; in equal volume with E via subcutaneous injection), daily for 4 weeks (n = 12/group). Estradiol or 17β-estradiol is a major circulating estrogen during active reproductive period in female and is the form of estrogen that exerts most potent estrogenic effect^48^. In this study, 17β-estradiol powder (Sigma-Aldrich Co., MO, USA) was dissolved in ethanol (1 mg/ml), then the solution was mixed with sesame oil (Sigma-Aldrich Co., MO, USA) before subcutaneously injected to rats (i.e. NDOE and HFOE rats). The dose of estradiol and vildagliptin used in this study had been demonstrated to improve cardiometabolic function in obese-insulin resistant rats^38^,^49^. Moreover, these selected doses were also similar to the doses used in clinical setting (transdermal estrogen 25–100 μg/day; vildagliptin 1–2 mg/kgBW/day)^7^,^50^. Body weight and food intake of all rats were recorded throughout the experimental period. After 4 weeks of treatment, post-treatment data from echocardiography and HRV were examined. Six-hour fasting was assigned to all rats then the fasted blood samples were collected from tail vein, and were centrifuged for plasma preparation. Then, the cardiac I/R procedure was performed. After I/R study, the heart was removed in order to study cardiac mitochondrial function and biochemical activities.

### Ovariectomy

In the ovariectomized group, rats were anesthetized with Xylazine (0.15 ml/kg) and Zolitil (50 mg/kg)^15^. After hair shaving and skin cleaning, a bilateral ovariectomy was carried out by initially making a midline dorsal skin incision. The incision was centered between the inferior crest of the rib cage and superior base of the thigh. The abdominal-pelvic cavity was accessed then the uterine tubes and ovaries were identified. Both ovaries were removed and uterine horns were returned into the cavity. In a sham group, all rats received the same anesthesia and also the same surgical preparation procedures as OVX rats. Bilateral ovaries of sham rats were identified and exposed, however no excision was done to the ovaries. After the operation, rats were individually housed in a clear box with dry bedding for 1 week before being randomized to be fed on a normal diet or high-fat diet.

### Echocardiography protocol

Echocardiography is a non-invasive method used for the assessment of left ventricular function. Rats were lightly anesthetized (2% Isoflurane with oxygen, via inhalation), then the chest was shaved and they were placed in a supine position. After echocardiography transmission gel was applied, an echocardiography probe (S12, Hewlett Packard) which was connected to an echocardiograph (SONOS4500, Philips), was used for collecting the data from the heart. Signals from M-mode echocardiography at the papillary muscle level were recorded. Parameters obtained from echocardiography were: 1) RVDd = right ventricular dimension during diastole; (2) IVSs,d = systolic and diastolic interventricular septum; (3) LVIDs,d = systolic and diastolic left ventricular internal dimension; and (4) LVPWs,d = left ventricular posterior wall thickness during systole and diastole. Fractional shortening (FS) and ejection fraction (EF) were calculated by the following formula; %FS = ((LVIDd − LVIDs)/LVIDd)*100, and % EF = ((LVEDV − LVESV)/LVEDV)*100^51^. After investigation, animals were allowed to fully recover and then returned to the cages.

### Heart rate variability (HRV) protocol

HRV is a non-invasive assessment of cardiac autonomic innervation activity. Rats were anesthetized by using isoflurane inhalation. In the prone position, needle electrodes were subcutaneously placed at the right arm, trunk and left leg of the animal. Electrocardiograms (ECG) were recorded in the animals using a signal transducer (PowerLab 4/25T, ADInstrument) and operated through Chart 5.0 program for 20 minutes consecutively^38^,^49^. During the recording of the ECG, the rat was in full conscious and was individually confined within a restrainer to limit the mobility. ECG data were then analyzed using the frequency-domain method by the MATLAB program to determine the high-frequency (HF) component (ranging between 0.15–0.40 Hz) and low-frequency (LF) component (ranging between 0.04–0.15 Hz)^15^,^52^. Cardiac sympathovagal control was reported as LF/HF ratio. Increased LF/HF ratio was used as an indication of cardiac sympathovagal imbalance^15^,^52^.

### Cardiac ischemia/Reperfusion study

After 4-weeks of treatment, a cardiac I/R study was performed on all rats. The rats were anesthetized and ventilated by tracheostomy with a positive pressure ventilator (Harvard rodent ventilator model 683, Harvard apparatus, Massachusetts, USA). ECG limb leads were placed to enable the recording of cardiac electrocardiogram and heart rate using a Powerlab signal transducer (Powerlab 4/25T, ADInsrument)^38^. A left-side thoracotomy incision at the 4^th^ intercostal space was made, and then the pericardium was incised to expose the pumping heart. The left anterior descending coronary artery (LAD) was identified and ligated at 2-mm distally to the origin using 5–0 silk suture. Cardiac ischemia was induced for 30 minutes and then followed by 120-minute reperfusion. Myocardial ischemia was indicated by the presence of ST elevation on ECG and the color changes at the ischemic area of myocardium. The time to 1^st^ VT/VF and mortality rate were investigated. Arrhythmia score was evaluated during the reperfusion period, using the previously described criteria^53^,^54^.

### Pressure-volume (P-V) loop study during cardiac I/R

Prior to the I/R protocol, rats were anesthetized by intramuscular injection with Zoletil (50 mg/kg, Vibbac Laboratories, Carros, France) and Xylazine (0.15 mg/kg, Laboratories Carlier, SA, Barcelona, Spain), then placed in the supine position^13^,^38^,^54^. Rats were ventilated with room air via a tracheostomy tube. The right carotid artery was identified and ligated, and then a pressure-volume (P-V) loop catheter (Scisence, Ontario, Canada) was inserted. The catheter tip was directed into the left ventricular chamber to record LV pressure and volume. After allowing 5 minutes for stabilization, the signaling data from the P-V loop catheter was recorded while the cardiac I/R protocol was performed. After I/R study, rats were sacrificed and their hearts were removed and prepared for further mitochondrial and biochemical studies. The investigated parameters obtained from the P-V loop study consisted of end-systolic pressure (ESP), end-diastolic pressure (EDP), maximum and minimum dP/dt (+dP/dt and −dP/dt), stroke volume (SV) and heart rate (HR). All P-V loop parameters were analyzed using Labscribe analytical software (Labscribe, Dover, NH, USA).

### Determination of myocardial infarct size in rat

At the end of the cardiac I/R experiment, blood sample was collected from the abdominal aorta for the examination of post I/R lactate dehydrogenase (LDH) level to identify the level of myocardial injury^55^. The plasma LDH level determination was performed by the clinical chemistry laboratory of the central diagnostic laboratory, Maharajnakorn Chiang Mai hospital, Chiang Mai University, Thailand. The heart was removed and irrigated with normal saline solution. LAD was re-occluded at the same site which had been previously ligated. Evan blue dye was infused into the heart through the catheter inserted at right and left coronary ostria. Non-blue-dyed areas were defined as no-blood flow areas or areas at risk (AAR). The heart was frozen and sliced horizontally from the base to the occluded area, into1-mm thick tissue slices. The tissue slices were immersed in Triphenyltetrazolium chloride (TTC) for about 12–15 minutes in order to turn the non-infarct or viable tissues to red color. Therefore, the area which exhibited neither blue nor red was identified as showing myocardial infarction^13^,^38^,^54^. Then myocardial infarct size was evaluated using Image tool software version 3.0.

### Cardiac mitochondrial function study

The protocols for cardiac mitochondrial function study, i.e. the mitochondrial ROS production, mitochondrial membrane potential changes and mitochondrial swelling, have been previously described^56^,^57^. The removed heart was re-occluded at LAD and perfused with cold normal saline, then cardiac tissue from non-ischemic (remote area, R) and ischemic areas (I) were identified. Both I and R tissue were separately homogenized and centrifuged to isolate the cardiac mitochondria^38^,^58^. The isolated cardiac mitochondria were stained using Dichlorohydrofluorescein diacetate (DCFDA) dye, and then the mitochondrial ROS level was measured using a fluorescent microplate reader (BioTek,Winooski, VT), set at an excitation wavelength of 485 nm with an emission wavelength of 530 nm^56^,^57^. The dye 5,5′,6,6′-tetrachloro-1,1′,3,3′-tetraethylbenzimidazolcarbocyanine iodide (JC-1) was utilized for the detection of mitochondrial membrane potential change. The monomer form of JC-1 was excited at the wavelength of 485 nm and the emission detected at 590 nm (green fluorescence), whereas the JC-1 aggregate form was excited at the wavelength of 485 nm and the emission detected at 530 nm (red fluorescence)^56^,^57^. The depolarization of the mitochondrial membrane was indicated by a decreased ratio of red/green fluorescence intensity^56^,^57^. Cardiac mitochondrial swelling was detected using a spectrophotometer at 540 nm, continuously for 30 minutes. The swelling of mitochondria was indicated by a decrease in the absorbance of a mitochondrial suspension. A transmission electron microscope (TEM; JEM-1200 EX II, JEOL Ltd., Japan) was used for projection the mitochondrial morphology, according to the previously described procedure^34^.

### Determination of oxidative stress

Oxidative stress levels in cardiac issue and serum were indicated by increased malondialdehyde (MDA) concentrations, which were measured using a high performance liquid chromatography (HPLC) system (Thermo Scientific, Bangkok, Thailand) as previously described^12^. Proteins from cardiac tissues, and serum were mixed with 10% trichloroacetic acid (TCA). The mixture was centrifuged and the supernatant was mixed with 0.44 M H3PO4 and 0.6% thiobabituric acid (TBA) solution to generate thiobarbituric acid reactive substances (TBARS), which were further measured by the HPLC system using BDS software (BarSpec Ltd., Rehovot, Israel). The concentration of TBARS was determined directly from a standard curve and reported as a MDA equivalent concentration^12^.

### Cardiac expression of Bax, Bcl-2, Cytochrome C and Connexin 43

The expressions of studied proteins, i.e. Bax, Bcl-2, cytochrome C and Connexin 43 (Cx43, in both phospho- and total forms) were determined by western blot analysis. After cardiac I/R protocol were completed, the unstained heart was identified for ischemic (I) area and remote (R) area, and then myocardial tissues were processed for protein extraction. The myocardial tissues from I and R areas were separately homogenized in a lysis buffer (containing 1% Nonidet P-40, 0.5% sodium deoxycholate, 0.1% sodium dodecyl sulfate (SDS) in 1xPBS) and were centrifuged at 13,000 rpm for 10 minutes. Myocardial protein was mixed with the loading buffer (consisting of 5% mercaptoethanol, 0.05% bromophenol blue, 75 nM Tris-HCl, 2% SDS and 10% glycerol with pH 6.8) at 1 mg/ml concentration and boiled at 95 °C for 5 minutes. The protein was loaded into a 10% SDS-polyacrylamide gel, and then transferred to a polyvinyldene difluoride (PVDF) membrane in a transfer system (Bio-Rad). The protein-containing membranes were incubated in 5% skimmed milk in 1xTBS-T buffer for 1 hour, and then exposed to anti-Bax, anti-Bcl-2, anti-phospho-Cn43 (Cell Signaling Technology, Danvers, MA, USA), anti-total-Cn43 (Santa Cruz Biotechnology, Santa Cruz, CA, USA), anti-cytochrome C and anti-actin (Sigma-Aldrich, St. Louis, MO, USA). Horseradish peroxidase conjugated with anti-rabbit or anti-mouse IgG (Santa Cruz Biotechnology, Inc., CA, USA) was administered to induce a peroxidase reaction, which further developed the signal by enhanced chemiluminescence (ECL) detection reagents. Finally, autoradiography was performed, and the immune-blotted films were investigated to examine the protein band density using the ImageJ analysis program (NIH image)^34^.

### Determination of metabolic parameters and hormone levels

Plasma was prepared from fasted blood samples and was kept frozen at −80 °C until analysis of glucose, cholesterol, triglyceride, insulin, estradiol and malondialdehyde (MDA) levels. Plasma estrogen concentration was measured by using a competitive enzyme immunoassay (EIA) kit (Cayman Chemical Company, MI, USA). Plasma insulin level was detected by sandwich ELISA kit (Millipore, MI, USA). Plasma glucose and triglyceride levels were determined by colorimetric assay from a commercially available kit (Biotech, Bangkok, Thailand). Fasting plasma HDL and LDL were determined using commercially available kits (ERBA diagnostic, Mannheim, Germany)^59^. Plasma lactate dehydrogenase (LDH) was determined by the clinical chemistry laboratory of the central diagnostic laboratory, Maharajnakorn Chiang Mai hospital, Chiang Mai University, Thailand.

### Chemicals and antibodies

Estradiol and sesame oil were obtained from Sigma-Aldrich (MO, USA). Vildagliptin was from Novartis (Thailand). Colorimetric assay kits for the determination of plasma glucose, cholesterol and triglycerides were from Biotech (Bangkok, Thailand). ELISA kit for the measurement of plasma insulin level was from Millipore (MI, USA). EIA kit for serum estradiol level detection was from Cayman Chemical Company (MI, USA). The antibodies against Bax, Bcl-2, cytochrome C and p-Cx43 were from Cell Signaling Technology (Danvers, MA, USA), total-Cx43 was from Santa Cruz Biotechnology (Santa Cruz, CA, USA), and anti-actin was from Sigma-Aldrich (MO, USA). The horseradish peroxidase conjugated with anti-rabbit and anti-mouse IgG was from Santa Cruz Biotechnology (Santa Cruz, CA, USA).

### Statistical analysis

All data is presented as mean ± standard error of mean (SEM). A one-way ANOVA followed by post-hoc Tukey’s test carried out using the SPSS program (SPSS version 16, SPSS Inc.) was used to determine the differences between the means. P value of less than 0.05 (P < 0.05) is considered as statistically significant.

### Limitation of the study

Since the treatments were delivered via different routes, different levels of stress and pharmacokinetic differences with different routes of administration could have affected metabolic outcomes. In this study, myocardial apoptosis markers were investigated (i.e. cardiac Bax expression, cardiac cytochrome C and plasma LDH level) and indicated the increased apoptosis in this study, however TUNEL assay was not performed. It has been known that high-fat diet consumption and estrogen deprivation cause an increased oxidative stress and cardiometabolic dysfunction^12^,^15^. We previously compared the effect of high-fat diet consumption (a high-fat diet sham group; HFS) and estrogen deprivation (normal-diet fed rats with ovariectomy; NDO) on plasma and cardiac oxidative stress, and our results indicated that both HFS and NDO had similar degree of oxidative stress and cardiometabolic dysfunction^15^. In that study, we also further demonstrated that plasma and cardiac oxidative stress levels were higher in high-fat fed rats with estrogen deprivation (HFO), compared to HFS and NDO rats^15^. In this study, we aimed to focus on the therapeutic strategy that could attenuate oxidative stress and cardiometabolic dysfunction only in estrogen deprived model subjected to ischemia/reperfusion injury. Since HFS and NDO exhibited similar effects on oxidative stress and cardiometabolic function, a non-estrogen deprived obese-insulin resistant (HFS) group was not included in this study. Although the reduction of oxidative stress level was proposed as an important mechanism of vildagliptin and estradiol in cardiometabolic function improvement, its impact was not directly measured in this study. The complete mechanisms regarding the potential effect of vildagliptin and estradiol still need the further investigation.

## Additional Information

**How to cite this article**: Sivasinprasasn, S. *et al*. DPP-4 Inhibitor and Estrogen Share Similar Efficacy Against Cardiac Ischemic-Reperfusion Injury in Obese-Insulin Resistant and Estrogen-Deprived Female Rats. *Sci. Rep.* **7**, 44306; doi: 10.1038/srep44306 (2017).

**Publisher's note:** Springer Nature remains neutral with regard to jurisdictional claims in published maps and institutional affiliations.

## Supplementary Material

Supplementary Information

## Figures and Tables

**Table 1:** Metabolic parameters of experimental groups at after 4 weeks of treatments.

Parameters	NDS	NDO			HFO		
		Ve	E	Vil	Ve	E	Vil
Body weight (g)	300 ± 14	338 ± 7*	304 ± 8^†^	333 ± 11*	393 ± 10^†^	351 ± 6^‡^	394 ± 8
Visceral fat (g)	14.7 ± 1.4	14.5 ± 1.7	11.2 ± 1.8	12.0 ± 1.0	30.8 ± 1.7^†^	20.5 ± 2.3^‡^	29.1 ± 2.9
Uterus weight (g)	0.38 ± 0.0	0.07 ± 0.0*	0.40 ± 0.0^†^	0.06 ± 0.0*	0.06 ± 0.0	0.36 ± 0.0^‡^	0.06 ± 0.0
Glucose (mg/dl)	130.7 ± 2.3	184.2 ± 3.5*	134.4 ± 3.9^†^	134.0 ± 3.9^†^	233.4 ± 4.5^†^	147.1 ± 4.6^‡^	148.5 ± 5.5^‡^
Insulin (ng/ml)	1.06 ± 0.0	2.40 ± 0.2*	1.17 ± 0.0^†^	1.16 ± 0.5^†^	2.68 ± 0.2	1.26 ± 0.0^‡^	1.23 ± 0.0^‡^
HOMA index	8.42 ± 0.4	21.36 ± 1.3*	9.13 ± 0.5^†^	8.97 ± 0.6^†^	29.93 ± 2.1^†^	11.33 ± 0.4^‡^	10.04 ± 0.7^‡^
Cholesterol (mg/dl)	70.4 ± 3.1	101.5 ± 4.12*	80.6 ± 4.6	78.4 ± 4.8	160.0 ± 5.0^†^	145.9 ± 8.0	145.1 ± 12.6
HDL (mg/dl)	41.7 ± 2.7	32.3 ± 1.7*	41.0 ± 1.8^†^	39.9 ± 1.7^†^	27.9 ± 2.4	41.0 ± 3.3^‡^	41.4 ± 4.1^‡^
LDL (mg/dl)	29.1 ± 2.1	53.2 ± 3.3*	30.9 ± 6.9^†^	32.9 ± 6.1^†^	114.4 ± 4.5^†^	86.2 ± 5.9^‡^	82.7 ± 16.8^‡^
Triglyceride (mg/dl)	50.1 ± 4.4	57.4 ± 5.9	51.2 ± 5.5	51.3 ± 4.3	59.0 ± 4.9	52.3 ± 3.9	52.1 ± 6.7
Estradiol level (pg/ml)	143 ± 5	53 ± 3*	196 ± 21*^†^	59 ± 6*	56 ± 8	202 ± 24^‡^	53 ± 11
Serum MDA (μmol/ml)	5.56 ± 0.2	6.18 ± 0.1*	5.42 ± 0.3^†^	5.31 ± 0.2^†^	6.28 ± 0.1	5.38 ± 0.2^‡^	5.40 ± 0.1^‡^
Tissue MDA (μmol/ml)	4.71 ± 1.1	7.76 ± 1.1*	4.48 ± 0.6^†^	3.85 ± 0.58^†^	7.94 ± 1.1	4.58 ± 0.4^‡^	4.63 ± 0.8^‡^
Food intake (gram/day)	15.6 ± 0.0	15.2 ± 0.3	14.2 ± 0.1	14.3 ± 0.3	18.6 ± 0.2^†^	18.6 ± 0.2	17.9 ± 0.5

Values are mean ± SEM. *P < 0.05 vs NDS, ^†^P < 0.05 vs NDOVe, and ^‡^P < 0.05 vs HFOVe. NDS, normal-diet fed sham-operated rats; NDO, normal-diet fed ovariectomized rats; HFO, high-fat-diet fed ovariectomized rats; Ve, vehicle; E, estradiol; Vil, vildagliptin; HOMA, Homeostasis Model Assessment; MDA, Malondialdehyde.

**Table 2:** Effect of estrogen and vildagliptin on cardiac function before I/R injury.

Parameters	NDS	NDO			HFO		
		Ve	E	Vil	Ve	E	Vil
Heart rate (bpm)	231 ± 23	249 ± 16	229 ± 19	231 ± 27	251 ± 12	234 ± 19	234 ± 26
LVESP (mmHg)	119 ± 12	73 ± 11*	99 ± 14	92 ± 8	75 ± 15	101 ± 18	102 ± 12
LVEDP (mmHg)	9 ± 2	20 ± 4*	7 ± 2	9 ± 3	21 ± 4	13 ± 1	15 ± 5
+dP/dt (mmHg/sec)	9328 ± 1617	4986 ± 1238*	6433 ± 672	6973 ± 791	4713 ± 1061	6391 ± 959	7023 ± 856
−dP/dt (mmHg/sec)	−6532 ± 1452	−2749 ± 216*	−4986 ± 1095	−4087 ± 560	−2812 ± 621	−4099 ± 688	−4283 ± 457
SV/BW (μl/gram)	1.02 ± 0.13	0.72 ± 0.10*	1.21 ± 0.05^†^	1.15 ± 0.06^†^	0.71 ± 0.08	1.09 ± 0.06^‡^	1.06 ± 0.05^‡^

Values are mean ± SEM (n = 6 per group). *P < 0.05 vs NDS, ^†^P < 0.05 vs NDOVe, and ^‡^P < 0.05 vs HFOVe. NDS, normal-diet fed sham-operated rats; NDO, normal-diet fed ovariectomized rats; HFO, high-fat-diet fed ovariectomized rats; Ve, vehicle; E, estradiol; Vil, vildagliptin; LVESP, left ventricular end systolic pressure; LVEDP, left ventricular end diastolic pressure; +dP/dt, maximal slope of the systolic pressure increment; −dP/dt, maximal slope of the diastolic pressure decrement; SV/BW, stroke volume/body weight.

**Table 3:** Effect of estrogen and vildagliptin on cardiac function at the end of myocardial ischemia.

Parameters	NDS	NDO			HFO		
		Ve	E	Vil	Ve	E	Vil
Heart rate (bpm)	218 ± 18	241 ± 20	205 ± 26	221 ± 29	236 ± 11	221 ± 12	224 ± 21
LVESP (mmHg)	92 ± 5	61 ± 9*	87 ± 4^†^	79 ± 2^†^	46 ± 5	95 ± 5^‡^	85 ± 17^‡^
LVEDP (mmHg)	17 ± 2	27 ± 4	20 ± 5	17 ± 5	30 ± 2	16 ± 1^‡^	16 ± 2^‡^
+dP/dt (mmHg/sec)	5478 ± 642	4154 ± 1472	5521 ± 1032	5258 ± 264	3974 ± 1062	5712 ± 1174	5544 ± 937
−dP/dt (mmHg/sec)	−4638 ± 1483	−1521 ± 522*	−3547 ± 563	−3126 ± 111	−1745 ± 635	−3802 ± 962	−3926 ± 850
SV/BW (μl/gram)	0.90 ± 0.05	0.48 ± 0.04*	1.04 ± 0.05^†^	0.85 ± 0.05^†^	0.30 ± 0.05^†^	0.53 ± 0.06^‡^	0.51 ± 0.09^‡^

Values are mean ± SEM (n = 6 per group). *P < 0.05 vs NDS, ^†^P < 0.05 vs NDOVe, and ^‡^P < 0.05 vs HFOVe. NDS, normal-diet fed sham-operated rats; NDO, normal-diet fed ovariectomized rats; HFO, high-fat-diet fed ovariectomized rats; Ve, vehicle; E, estradiol; Vil, vildagliptin; LVESP, left ventricular end systolic pressure; LVEDP, left ventricular end diastolic pressure; +dP/dt, maximal slope of the systolic pressure increment; −dP/dt, maximal slope of the diastolic pressure decrement; SV/BW, stroke volume/body weight.

**Table 4:** Effect of estrogen and vildagliptin on cardiac function at the end of reperfusion.

Parameters	NDS	NDO			HFO		
		Ve	E	Vil	Ve	E	Vil
Heart rate (bpm)	233 ± 21	273 ± 12	213 ± 33	228 ± 35	214 ± 5	217 ± 21	205 ± 26
LVESP (mmHg)	85 ± 10	48 ± 11*	79 ± 9^†^	78 ± 9^†^	41 ± 12	81 ± 12^‡^	82 ± 13^‡^
LVEDP (mmHg)	14 ± 2	26 ± 3*	12 ± 5^†^	10 ± 4^†^	32 ± 1	14 ± 2^‡^	12 ± 3^‡^
+dP/dt (mmHg/sec)	6283 ± 395	4511 ± 1311	5889 ± 87	5879 ± 1124	2586 ± 738	5682 ± 1280	5882 ± 1130
−dP/dt (mmHg/sec)	−4029 ± 1826	−2759 ± 967	−3817 ± 411	−3313 ± 506	−2962 ± 145	−3881 ± 360	−3967 ± 884
SV/BW (μl/gram)	0.68 ± 0.10	0.36 ± 0.03*	0.88 ± 0.04^†^	0.87 ± 0.10^†^	0.40 ± 0.07	0.64 ± 0.11^‡^	0.70 ± 0.10^‡^

Values are mean ± SEM (n = 6 per group). *P < 0.05 vs NDS, ^†^P < 0.05 vs NDOVe, and ^‡^P < 0.05 vs HFOVe. NDS, normal-diet fed sham-operated rats; NDO, normal-diet fed ovariectomized rats; HFO, high-fat-diet fed ovariectomized rats; Ve, vehicle; E, estradiol; Vil, vildagliptin; LVESP, left ventricular end systolic pressure; LVEDP, left ventricular end diastolic pressure; +dP/dt, maximal slope of the systolic pressure increment; −dP/dt, maximal slope of the diastolic pressure decrement; SV/BW, stroke volume/body weight.
