# Supplementary material for: DPP-4 Inhibitor and Estrogen Share Similar Efficacy Against Cardiac Ischemic-Reperfusion Injury in Obese-Insulin Resistant and Estrogen-Deprived Female Rats
Source: Sci Rep. 2017 Mar 10;7:44306. doi: 10.1038/srep44306 (PMC5345038; doi:10.1038/srep44306)
Supplement: Supplementary Information [file srep44306-s1.pdf]

Supplementary Information file

Title: DPP-4 Inhibitor and Estrogen Share Similar Efficacy Against Cardiac Ischemic-Reperfusion Injury in Obese-Insulin Resistant and Estrogen-Deprived Female Rats

Authors: Sivaporn Sivasinprasasn, Pongpan Tanajak, Wanpitak Pongkan, Wasana Pratchayasakul, Siriporn C. Chattipakorn, Nipon Chattipakorn

Supplementary figure legends:

Supplementary figure 1. Metabolic parameters of control, vehicle-, estrogen-and vildagliptin-treated groups After 4 weeks of treatments, body weight (a), fasting levels of cholesterol (b), HDL (c), glucose (d), insulin (e), HOMA index (f), serum MDA (h) and tissue MDA (I) were improved. Serum estradiol level (g) was increased in estrogen-treated rats. Values are expressed as mean ± SEM. *P <0.05 vs NDS, †P <0.05 vs NDOVe, and ‡P <0.05 vs HFOVe. NDS, normal-diet fed sham-operated rats; NDO, normal-diet fed ovariectomized rats; HFO, high-fat-diet fed ovariectomized rats; Ve, vehicle; E, estradiol; Vil, vildagliptin; HOMA, Homeostasis Model Assessment; MDA, Malondialdehyde.

Supplementary figure 2. Effect of estrogen and vildagliptin on cardiac contractile function The level of LVESP, LVEDP and SV/BW were evaluated at baseline (a), at the end of 30-minute ischemia (b), and at the end of 120-minute reperfusion (c). Values are mean ± SEM (n = 6 per group). *P <0.05 vs NDS, †P <0.05 vs NDOVe, and ‡P <0.05 vs HFOVe. NDS, normal-diet fed sham-operated rats; NDO, normal-diet fed ovariectomized rats; HFO, high-fat-diet fed ovariectomized rats; Ve, vehicle; E, estradiol; Vil, vildagliptin; BP, blood pressure; LVESP, left ventricular end systolic pressure; LVEDP, left ventricular end diastolic pressure; SV/BW, stroke volume/body weight.

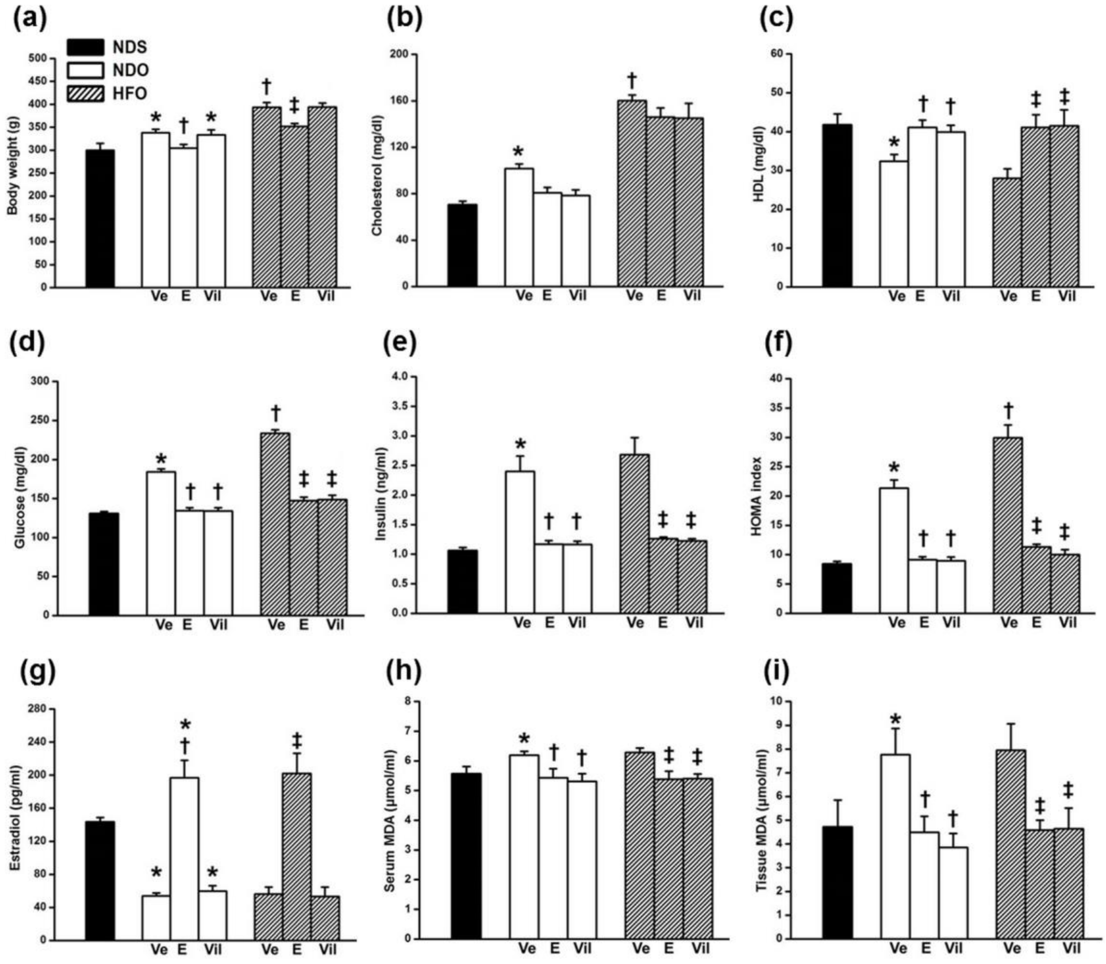
(a)
NDS
NDO
HFO
Body weight (g)
Ve E Vil Ve E Vil
(b)
Cholesterol (mg/dl)
Ve E Vil Ve E Vil
(c)
HDL (mg/dl)
Ve E Vil Ve E Vil
(d)
Glucose (mg/dl)
Ve E Vil Ve E Vil
(e)
Insulin (ng/ml)
Ve E Vil Ve E Vil
(f)
HOMA index
Ve E Vil Ve E Vil
(g)
Estradiol (pg/ml)
Ve E Vil Ve E Vil
(h)
Serum MDA (μmol/ml)
Ve E Vil Ve E Vil
(i)
Tissue MDA (μmol/ml)
Ve E Vil Ve E Vil

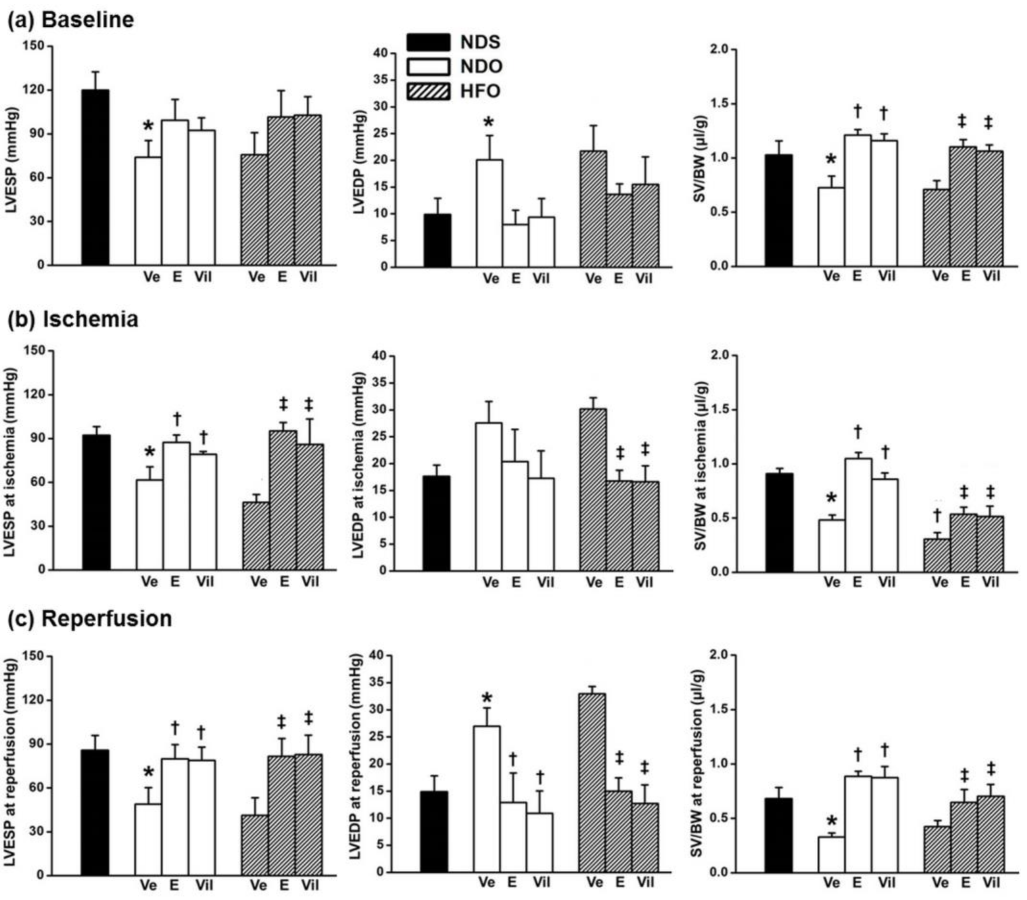

(a) Baseline
NDS
NDO
HFO
LVESP (mmHg)
LVEDP (mmHg)
SV/BW (µl/g)
Ve E Vil Ve E Vil
(b) Ischemia
LVESP at ischemia (mmHg)
LVEDP at ischemia (mmHg)
SV/BW at ischemia (µl/g)
(c) Reperfusion
LVESP at reperfusion (mmHg)
LVEDP at reperfusion (mmHg)
SV/BW at reperfusion (µl/g)
